# Accuracy Verification of a Computed Tomography-Based Navigation System for Total Hip Arthroplasty in Severe Hip Dysplasia: A Simulation Study Using 3D-Printed Bone Models of Crowe Types II, III, and IV

**DOI:** 10.3390/medicina61060973

**Published:** 2025-05-24

**Authors:** Ryuichiro Okuda, Tomonori Tetsunaga, Kazuki Yamada, Tomoko Tetsunaga, Takashi Koura, Tomohiro Inoue, Yasutaka Masada, Yuki Okazaki, Toshifumi Ozaki

**Affiliations:** 1 Department of Orthopaedic Surgery, Okayama University Graduate School of Medicine, Dentistry and Pharmaceutical Sciences, Okayama 700-8558, Japan; ryuichiro.okuda@gmail.com (R.O.);; 2 Department of Musculoskeletal Health Promotion, Faculty of Medicine, Dentistry and Pharmaceutical Sciences, Okayama University, Okayama 700-8558, Japan; 3 Department of Orthopaedic Surgery, Okayama University Hospital, Okayama 700-8558, Japan; 4 Department of Sports Medicine, Faculty of Medicine, Dentistry and Pharmaceutical Sciences, Okayama University, Okayama 700-8558, Japan; 5 Center for Education in Medicine and Health Sciences, Okayama University, Okayama 700-8558, Japan; 6 Department of Orthopaedic Surgery, Faculty of Medicine, Dentistry and Pharmaceutical Sciences, Okayama University, Okayama 700-8558, Japan

**Keywords:** total hip arthroplasty, CT-based navigation, bone model, artificial intelligence, Ortoma Treatment Solution

## Abstract

*Background and Objective*: The use of computed tomography (CT)-based navigation systems has been shown to improve surgical accuracy in total hip arthroplasty. However, there is limited literature available about the application of CT-based navigation systems in severe hip dysplasia. This study aimed to evaluate the accuracy of a CT-based navigation system in patients with severe hip dysplasia using three-dimensional (3D)-printed bone models. *Methods*: 3D-printed bone models were generated from CT data of patients with severe hip dysplasia (Crowe type II, 10 hips; type III, 10 hips; and type IV, 10 hips). The accuracy of automatic segmentation, success rate, point-matching accuracy across different registration methods, and deviation values at reference points after registration were assessed. *Results*: For the combined cohort of Crowe II, III, and IV cases (*n* = 30), the Dice Similarity Coefficient and Jaccard Index were 0.99 ± 0.01 and 0.98 ± 0.02, respectively. These values indicate a high level of segmentation accuracy. The “Matching with true and false acetabulum + iliac crest” method achieved a 100% success rate across all groups, with mean deviations of 0.08 ± 0.28 mm in the Crowe II group, 0.12 ± 0.33 mm in the Crowe III group, and 0.14 ± 0.50 mm in the Crowe IV group (*p* = 0.572). In the Crowe IV group, the anterior superior iliac spine deviation was significantly lower using the “Matching with true and false acetabulum + iliac crest” method compared to the “Matching with true and false acetabulum” method (0.28 ± 0.49 mm vs. 3.29 ± 2.56 mm, *p* < 0.05). *Conclusions*: This study demonstrated the high accuracy of automatic AI-based segmentation, with a Dice Similarity Coefficient of 0.99 ± 0.01 and a Jaccard Index of 0.98 ± 0.02 in the combined cohort of Crowe type II, III, and IV cases (*n* = 30). The matching success rate was 100%, with additional points on the iliac crest, which improved matching accuracy and reduced deviations, depending on the case.

## 1. Introduction

The clinical course of total hip arthroplasty (THA) has demonstrated excellent treatment outcomes, including reliable pain relief, superior functional recovery, reduced complications such as postoperative dislocation, and an implant survival rate of nearly 90% at 30 years postoperatively [1,2,3,4]. Correct implant component positioning within the safe zone during THA is crucial for minimizing the risk of postoperative complications, including dislocation, reduced range of motion, accelerated wear, and early loosening [5,6,7,8,9,10,11,12]. Currently, available computer-assisted surgical tools have been shown to improve surgical accuracy in THA, thereby enhancing surgical outcomes and reducing the need for revision hip surgery [13,14,15,16].

THA in patients with developmental dysplasia of the hip (DDH) presents significant challenges due to complex acetabular and femoral anatomy [17]. In high-grade DDH, the acetabulum is typically triangular, widens distally, and has a thick posterior wall [18]. CT-based navigation has emerged as a valuable tool to improve the accuracy and reproducibility of component positioning in THA for patients with DDH; however, limited literature is available on the use of CT-based navigation systems in cases of severe hip dysplasia [19].

Recent studies have demonstrated the utility of artificial intelligence (AI)-based preoperative planning systems in THA, showing superior accuracy in predicting prosthesis size and positioning compared to traditional methods [20,21]. The Ortoma Treatment Solution (OTS, Ortoma AB, Gothenburg, Sweden) is a novel computer-assisted AI-based orthopedic surgical platform [22]. A distinguishing feature of the OTS, compared to other computer-assisted tools, is its integration with advanced AI algorithms and machine learning models. Unlike robotic-assisted THA systems, which require bulky equipment and intraoperative tracking, OTS is a CT-based navigation system that assists with both preoperative planning and intraoperative guidance. While robotic systems offer high accuracy, they are often limited by high costs, longer setup times, and increased surgical invasiveness. In contrast, OTS provides a less invasive and more streamlined approach, potentially making it more accessible and practical for routine clinical use. While the outcomes of OTS have been previously reported in patients with primary hip osteoarthritis [22], its application in patients with severe hip dysplasia has not been reported.

The main objective of this study was to assess the accuracy of a CT-based navigation system with AI-based algorithms (OTS) in patients with severe hip dysplasia. Specifically, we aimed to evaluate the accuracy of segmentation by OTS using computed tomography (CT) data from patients with severe hip dysplasia (Crowe types II, III, and IV) [23], the precision of navigation matching with three-dimensional (3D)-printed bone models, and the deviation after matching.

## 2. Materials and Methods

### 2.1. Study Design

A 3D-printed bone model was used to evaluate the CT-based navigation system with AI-based algorithms (OTS). The study protocol was approved by the institutional ethics committee (registration number: 2310-016). Informed consent was obtained through an opt-out option on the website.

**Figure 1:**
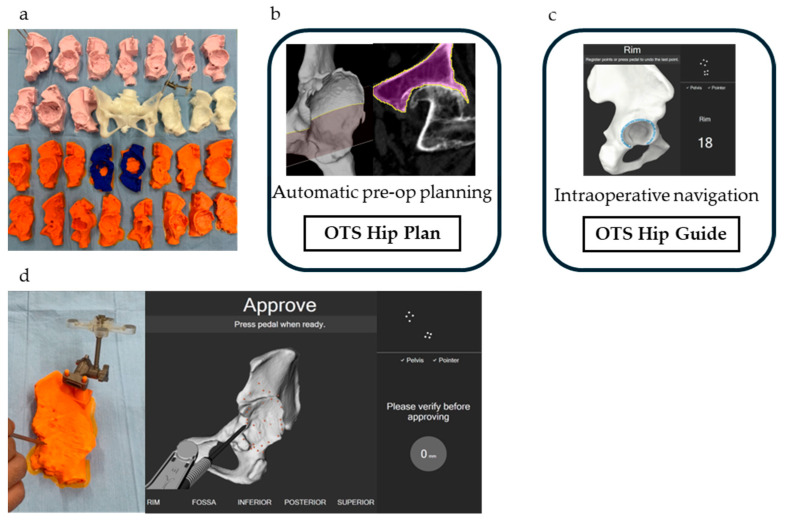
(**a**) 3D-printed bone models of 30 hips; (**b**) Automatic computer-assisted preoperative 3D planning using the OTS Hip Plan; (**c**) Surgeon-controlled navigation during surgery with the OTS Hip Guide; (**d**) Application of the OTS to 3D-printed bone models.

Preoperative CT scans were retrieved from the hospital medical imaging database for patients who underwent THA by December 2024. The inclusion criteria were a diagnosis of severe hip dysplasia (Crowe types II, III, and IV) and CT scans of sufficient quality for 3D reconstruction. CT data with 1 mm slice thickness were imported into ZedView (LEXI, Tokyo, Japan) to generate 3D bone models. Segmentation was performed using ZedEdit (LEXI, Tokyo, Japan), and the resulting models were refined to ensure anatomical accuracy. The generated 3D bone model data were exported in a standard triangulated language format and printed using a poly-L-lactic acid filament with AnkerMake Studio (Anker, Changsha, China) and AnkerMake M5 3D (Anker, Changsha, China) (Figure 1a). The AnkerMake M5 provides a dimensional accuracy of ±0.1 mm and a layer resolution of 0.2 mm.

CT scans in Digital Imaging and Communications in Medicine (DICOM) format were imported to the server using the in-built importer tool “OTS Patient import.” The imported DICOM data were processed automatically by the software “OTS Hip Plan” using AI (Figure 1b), and segmentation of the pelvis and femur was typically completed within a minute. After verifying the accuracy of automatic segmentation, the surgical plan data were imported into the ORTOMA Hip Guide (Figure 1c), where segmentation relative to the 3D-printed bone model was performed (Figure 1d). In cases where errors were found in the automatic segmentation, manual correction was performed to refine the segmentation.

**Figure 2:**
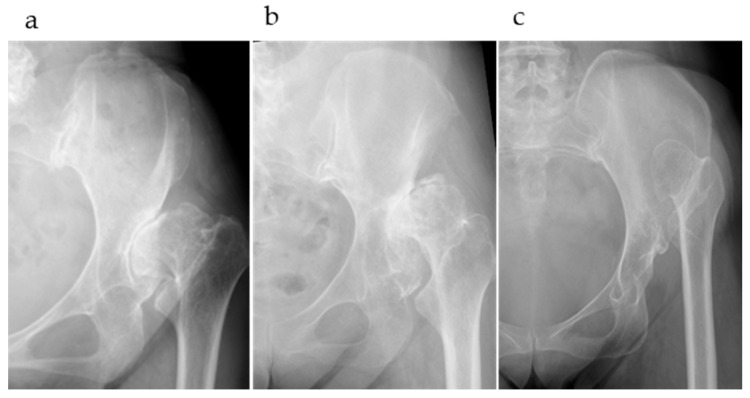
(**a**) Crowe type II, Hartofilakidis type B; (**b**) Crowe type III, Hartofilakidis type B; (**c**) Crowe type IV, Hartofilakidis type C.

Patients were divided into three groups based on the Crowe classification (types II, III, and IV; Figure 2). The following three evaluation items were examined: (1) the accuracy of automatic AI segmentation, (2) the success rate and accuracy of surface matching using three matching methods, and (3) deviation values at the reference points after surface matching, including a comparison of deviation values across different matching methods in Crowe type IV cases.

The accuracy of automatic AI-based segmentation was evaluated by comparing STL models automatically generated by the OTS with manually corrected STL models. Comparisons were performed using Python (version 3.13.3), following the segmentation evaluation approach described above. Specifically, both STL models were aligned using Iterative Closest Point (ICP) registration and then converted into voxel-based 3D binary masks within a shared bounding box. Evaluation metrics included the Dice Similarity Coefficient and Jaccard Index, which quantified spatial agreement between the automatically segmented and manually corrected models.

**Figure 3:**
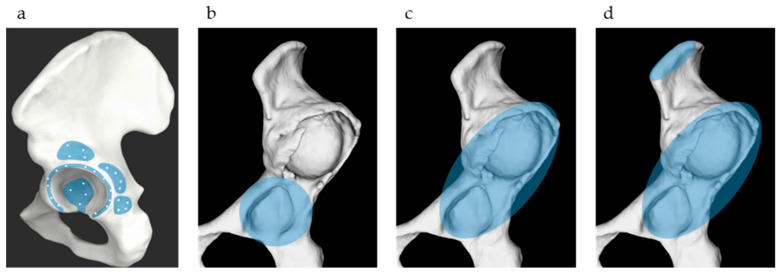
Three surface matching methods. (**a**) The light blue area indicates the recommended point acquisition zone by the OTS Hip Guide; (**b**) Matching with the true acetabulum; (**c**) Matching with the true and false acetabulum; (**d**) Matching with the true and false acetabulum + iliac crest.

The success rate and accuracy of surface matching were evaluated using three matching methods: (a) matching with the true acetabulum, where 34 points around the true acetabulum were matched based on the ORTOMA Hip Guide instructions; (b) matching with the true and false acetabulum, where 34 points around the true and false acetabulum were matched according to the instructions of the ORTOMA Hip Guide; and (c) matching with the true and false acetabulum + iliac crest, where 32 points around the true and false acetabula were matched along with two additional points on the iliac crest (Figure 3). In actual surgical practice, two 4 mm navigation pins are inserted into the iliac crest through a small incision. Therefore, acquiring two registration points on the iliac crest via the same incision is clinically feasible and is already being implemented in routine procedures. Deviations at five reference points, the anterior, posterior, superior, and inferior rims of the acetabulum, as well as the anterior superior iliac spine (ASIS), were measured. The mean deviation was calculated by averaging the deviations of the five reference points. Successful surface matching was determined based on the ORTOMA Hip Guide successfully completing the matching process and achieving a mean deviation of 2 mm or less.

Deviation values at the five reference points after matching were evaluated in the Crowe IV group to examine the differences in deviations between matching with the true and false acetabulum and matching with the true and false acetabulum + iliac crest.

### 2.2. Statistical Analysis

A univariate analysis was conducted to compare age, sex, body height, body weight, Hartofilakidis classification, segmentation accuracy, matching success rate, and mean deviation of the five reference points across the Crowe II, III, and IV groups [24,25]. Normally distributed variables were compared using analysis of variance (ANOVA), while categorical variables were analyzed using Fisher’s exact test. The Bonferroni method was applied for post hoc comparisons wherein significant differences were detected by ANOVA or Fisher’s exact test. For the seven successfully matched cases in the Crowe IV group using the true and false acetabulum method, deviations at the five reference points (anterior rim, posterior rim, superior rim, inferior rim, and ASIS) were compared between the true and false acetabulum and the true and false acetabulum + iliac crest methods using a paired *t*-test. The normality of continuous variables was assessed using the Shapiro–Wilk test. Statistical analyses were performed using EZR software (Saitama Medical Center, Jichi Medical University, Saitama, Japan) [26], with statistical significance set at *p* < 0.05.

## 3. Results

A total of 26 patients with 30 hips were analyzed: Crowe II group, 10 hips; Crowe III group, 10 hips; and Crowe IV group, 10 hips (Table 1). Both hips from the same patient were included in two cases. The Hartofilakidis Classification was A: 4 and B: 6 in the Crowe II group, A: 1 and B: 9 in the Crowe III group, and B: 4 and C: 6 in the Crowe IV group. No statistically significant differences were observed in age, sex, body height, body weight, body mass index, or hip side among the three groups. Statistically significant differences were observed in the Hartofilakidis classification between the Crowe II and IV groups (*p* < 0.05) and the Crowe III and IV groups (*p* < 0.05).

The accuracy of automatic AI-based segmentation was evaluated by comparing STL models automatically generated by the OTS with manually corrected STL models. For the combined cohort of Crowe II, III, and IV cases (*n* = 30), the Dice Similarity Coefficient and Jaccard Index were 0.99 ± 0.01 and 0.98 ± 0.02, respectively, indicating a high level of segmentation accuracy (Table 2). In the Crowe II group (*n* = 10), automatic segmentation achieved perfect agreement with the manually corrected models. The Dice Similarity Coefficient and Jaccard Index were both 1.00, indicating complete overlap. In the Crowe III group (*n* = 10), the Dice Similarity Coefficient and Jaccard Index were 0.99 ± 0.01 and 0.98 ± 0.03, respectively. In the Crowe IV group (*n* = 10), the Dice Similarity Coefficient and Jaccard Index were 0.99 ± 0.01 and 0.98 ± 0.02, respectively.

The matching success rate using the “Matching with true acetabulum” method was 50% in the Crowe II group, 30% in the Crowe III group, and 20% in the Crowe IV group (*p* = 0.310, Table 3). The mean deviation among the successfully matched cases was 0.33 ± 0.28 mm in the Crowe II group, 0.44 ± 0.45 mm in the Crowe III group, and 0.50 ± 0.51 mm in the Crowe IV group (*p* = 0.089). The matching success rate using the “Matching with true and false acetabulum” method was 100%, 80%, and 70% in the Crowe II, III, and IV groups, respectively (*p* = 0.321). The mean deviation among the successfully matched cases was 0.08 ± 0.14 mm in the Crowe II group, 0.34 ± 0.21 mm in the Crowe III group, and 0.68 ± 0.45 mm in the Crowe IV group (*p* < 0.001). A statistically significant difference was observed between the Crowe II and Crowe IV groups (*p* < 0.001). The matching success rate using the “Matching with true and false acetabulum + iliac crest” method was 100% in all groups, including Crowe II, III, and IV (*p* = 1.0). The mean deviation among the successfully matched cases was 0.08 ± 0.28 mm in the Crowe II group, 0.12 ± 0.33 mm in the Crowe III group, and 0.14 ± 0.50 mm in the Crowe IV group (*p* = 0.572).

**Figure 4:**
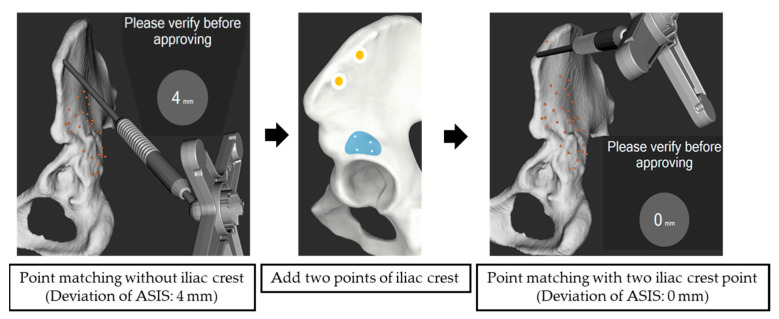
Surface matching with and without the iliac crest. The ASIS deviation was 4 mm without the iliac crest points and 0 mm with two iliac crest points.

In the Crowe IV group, deviations between the “Matching with true and false acetabulum” and “Matching with true and false acetabulum + iliac crest” methods were analyzed in seven hips that were successfully matched using the true and false acetabulum method. No significant differences were observed in deviations between the two methods for the anterior, posterior, superior, and inferior rims (Table 4). However, at the ASIS, the deviation was significantly lower with the “Matching with true and false acetabulum + iliac crest” method compared to the “Matching with true and false acetabulum” method (0.28 ± 0.49 mm vs. 3.29 ± 2.56 mm, *p* < 0.05; Figure 4).

## 4. Discussion

This study demonstrated the high accuracy of automatic AI-based segmentation, with a Dice Similarity Coefficient of 0.99 ± 0.01 and a Jaccard Index of 0.98 ± 0.02 in the combined cohort of Crowe type II, III, and IV cases (*n* = 30). These values indicate a very high level of segmentation accuracy, even in patients with severe hip dysplasia (Crowe types II, III, and IV). For registration, the matching success rate was 100% (30/30) when additional points on the iliac crest were acquired. The inclusion of additional points on the iliac crest improved the accuracy of ASIS deviation. This is the first report of the evaluation of the accuracy of the OTS in cases of severe hip dysplasia.

In general, preoperative planning has been associated with reducing surgical complications, improving surgical accuracy [27], minimizing implant inventory [28], and lowering costs [29]. Navigation systems provide real-time visualization and intraoperative guidance, mitigating the risk of implant misplacement and the need for revision surgeries. The OTS, an AI-based platform, assists surgeries throughout the THA process, including preoperative planning, intraoperative navigation, and postoperative analysis. In previous reports, the mean errors in patient surgeries were reported to be −0.07 ± 2.72° (ranging from −4.68 to 5.22) for anteversion, −0.2 ± 0.86° (ranging from −1.29 to 1.36) for inclination, and 0.28 ± 0.78 mm (ranging from −1.53 to 1.46) for cup depth [22]. It was concluded that the navigation tool provided intraoperative guidance, with remarkable precision in cup placement, effectively reducing the risk of cup malpositioning outside the patient-specific safe zone.

In this study, segmentation accuracy for severe hip dysplasia was very high, with segmentation completed within approximately one minute of import, contrasting with conventional CT-based navigation systems that require multiple manual steps such as bone segmentation, anatomical landmark localization, and alignment [22,30]. These cumbersome processes pose challenges by extending the patients’ preoperative waiting times and increasing the surgeons’ preparation times. The implementation of AI technology in THA assists surgeons in making clinical decisions, providing patient-specific planning, and improving surgical outcomes. Studies have shown that AI-based algorithms in arthroplasty have the potential to enhance patient care through better screening, planning, operating, and monitoring [31,32]. The ability to perform accurate and rapid preoperative planning, even in cases of severe hip dysplasia with significant deformities, is considered a major advantage.

In cases of severe hip dysplasia (Crowe III and IV cases), additional points on the iliac crest during OTS surface matching improved surface matching accuracy. These cases presented significant hip joint deformities, including shallow flat acetabulum, an external superior bone defect, an increased anterior tilt angle, pseudo-acetabulum formation, and a decrease in the diameter of the proximal femoral medullary cavity [33,34,35]. The need for additional matching points was particularly evident in cases such as Crowe type IV and Hartofilakidis type C (high dislocation), where the acetabulum was typically flat and lacked sufficient bony contours for accurate 3D point acquisition. Acquiring points in areas distant from the acetabulum, such as the iliac crest, in a 3D manner proved crucial for improving matching accuracy and reducing deviation. Previous studies have reported that adding points to the area orthogonal to the direction to be corrected improves correction ability [36].

The iliac crest was selected as an additional surface-matching region because of its anatomical distance from the acetabulum—minimizing the impact of local deformities—and its accessibility due to proximity to the skin surface. Importantly, surface points on the iliac crest can be reliably acquired even in patients with severe hip dysplasia. This approach may also be applicable to other complex hip deformities, supporting its utility as a versatile and practical solution to improve registration accuracy in challenging cases.

One limitation of this study is the use of 3D-printed bone models. While previous studies have employed such models in CT-based navigation validation, they do not replicate intraoperative factors such as patient positioning, soft tissue interference, or image distortion [36]. Although standard 3D printing technology was used for model fabrication in this study, recent advances in bioprinting may provide future opportunities for clinical integration, particularly in tissue engineering and patient-specific modeling. Compatibility with bioprinting platforms and materials, as previously suggested [37], warrants further investigation. Additional limitations include the inability to evaluate cup placement accuracy and clinical outcomes, since this study did not involve actual surgical procedures. Clinical outcomes and potential complications could not be assessed, and a formal power analysis was not performed due to the limited sample size. Moreover, both hips from the same patient were included in two cases, which may compromise the statistical independence of observations. However, given the small number of such cases, it likely has a minimal influence on the findings. While the iliac crest landmark appeared to enhance matching accuracy, its accessibility may be limited intraoperatively in patients with obesity or distorted anatomy. Finally, the study did not include a direct comparison between the OTS system and other navigation platforms; thus, conclusions regarding the superiority of the OTS system remain speculative. Future studies should investigate the clinical application of OTS in patients with severe hip dysplasia and assess procedural outcomes.

**Figure 5:**
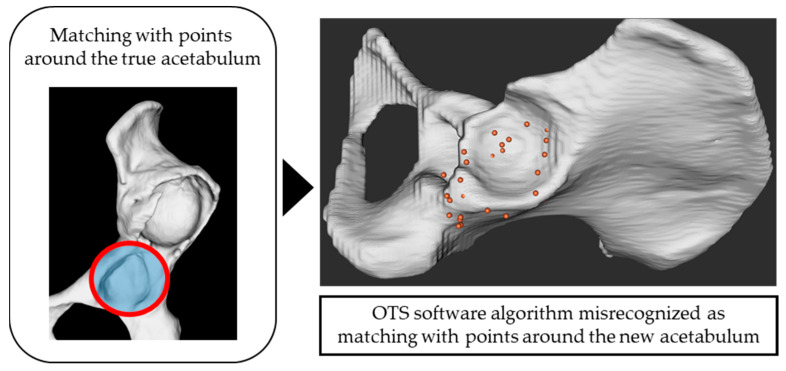
Although the points were collected around the true acetabulum, the OTS software algorithm mistakenly recognized them as being from the region around the new acetabulum.

In cases involving both the true and false acetabulum, collecting registration points only around the true acetabulum resulted in reduced matching: from 100% to 50% in the Crowe II group, 80% to 30% in the Crowe III group, and 70% to 20% in the Crowe IV group. This outcome likely stemmed from the OTS software algorithm misidentifying the collected points as corresponding to the false acetabulum after mechanical processing, despite their correct collection around the true acetabulum (Figure 5). This limitation highlights the need to expand point acquisition beyond the true acetabulum to include a broader and more 3D range, such as areas around the false acetabulum and iliac crest, to improve surface matching accuracy.

Further research should focus on directly comparing the performance of the OTS with that of other advanced navigation systems. Such studies could elucidate the unique advantages of the OTS, particularly regarding segmentation speed and accuracy. Additionally, further investigation into the correlation between the number and spatial distribution of collected points and resulting matching accuracy could inform and refine point acquisition strategies for complex cases.

## 5. Conclusions

This study demonstrated the high segmentation accuracy of a novel CT-based navigation system with AI-based algorithms for severe hip dysplasia, even for Crowe types II, III, and IV. For registration, the matching success rate was 100%, with the inclusion of additional points on the iliac crest, improving the matching success rate and accuracy. These findings highlight the potential of this novel CT-based navigation system for accurate preoperative planning in patients with severe hip dysplasia.

## Figures and Tables

**Table 1:** Patient demographics.

	Crowe II Group (*n* = 10)	Crowe III Group (*n* = 10)	Crowe IV Group (*n* = 10)	*p*-Value
Age (years)	60.6 ± 6.7	66.3 ± 7.4	61.0 ± 6.4	0.137
Sex (female/male)	7/3	7/3	9/1	0.195
Hip side (left/right)	6/4	5/5	7/3	0.709
Body height (cm)	157.7 ± 8.0	151.8 ± 7.2	150.2 ± 7.5	0.078
Body weight (kg)	58.8 ± 18.8	60.0 ± 6.4	50.4 ± 12.4	0.255
Body mass index (kg/m^2^)	23.2 ± 5.7	25.9 ± 2.4	22.2 ± 4.1	0.159
Hartofilakidis Classification (A/B/C)	4/6/0	1/9/0	0/4/6	<0.001

Hartofilakidis Classification, A: Dysplasia: the femoral head is contained within the original acetabulum despite the degree of subluxation. B: Low dislocation: the femoral head articulates with a false acetabulum, which partially covers the true acetabulum to a varying degree. C: High dislocation: the femoral head is completely out of the true acetabulum and migrated superiorly and posteriorly to a varying degree. Data are presented as means ± SD.

**Table 2:** Segmentation accuracy.

	Crowe II Group (*n* = 10)	Crowe III Group (*n* = 10)	Crowe IV Group (*n* = 10)	Total (*n* = 30)
Dice Similarity Coefficient (from 1.00 to 0)	1.00 ± 0.00	0.99 ± 0.01	0.99 ± 0.01	0.99 ± 0.01
Jaccard Index (from 1.00 to 0)	1.00 ± 0.00	0.98 ± 0.02	0.98 ± 0.03	0.98 ± 0.02

Data are presented as means ± SD.

**Table 3:** Surface matching rate and the deviation of the five reference points.

	Crowe II Group (*n* = 10)	Crowe III Group (*n* = 10)	Crowe IV Group (*n* = 10)	*p*-Value
Matching with true acetabulum				
Matching success rate (%)	50	30	20	0.310
Mean deviation of the five reference points (mm)	0.33 ± 0.28	0.44 ± 0.45	0.50 ± 0.51	0.089
Matching with true and false acetabulum				
Matching success rate (%)	100	80	70	0.321
Mean deviation of the five reference points (mm)	0.08 ± 0.14	0.34 ± 0.21	0.68 ± 0.45	<0.05
Matching with true and false acetabulum + iliac crest				
Matching success rate (%)	100	100	100	1.0
Mean deviation of the five reference points (mm)	0.08 ± 0.28	0.12 ± 0.33	0.14 ± 0.50	0.572

Data are presented as means ± SD.

**Table 4:** Deviation in the Crowe IV group.

	Matching with True and False Acetabulum (*n* = 7)	Matching with True and False Acetabulum + Iliac Crest (*n* = 7)	*p*-Value
Anterior rim (mm)	0	0.14 ± 0.38	0.356
Posterior rim (mm)	0.14 ± 0.38	0.28 ± 0.49	0.356
Superior rim (mm)	0	0.14 ± 0.38	0.356
Inferior rim (mm)	0.14 ± 0.38	0.14 ± 0.38	1.0
ASIS (mm)	3.29 ± 2.56	0.28 ± 0.49	<0.05

ASIS, anterior superior iliac spine. Data are presented as means ± SD.

## Data Availability

The original contributions presented in this study are included in the article. Further inquiries can be directed to the corresponding author.

## Abbreviations

The following abbreviations are used in this manuscript:

AI	artificial intelligence
ANOVA	analysis of variance
ASIS	anterior superior iliac spine
CT	computed tomography
DICOM	Digital Imaging and Communications in Medicine
OTS	Ortoma Treatment Solution
THA	total hip arthroplasty
